# Adoption of the Robotic Platform across Thoracic Surgeries

**DOI:** 10.3390/jcm13195764

**Published:** 2024-09-27

**Authors:** Kaity H. Tung, Sai Yendamuri, Kenneth P. Seastedt

**Affiliations:** 1 Department of Surgery, University at Buffalo, Buffalo, NY 14203, USA; kaitytun@buffalo.edu; 2 Department of Thoracic Surgery, Roswell Park Comprehensive Cancer Center, Buffalo, NY 14203, USA; sai.yendamuri@roswellpark.org

**Keywords:** robotic surgery, lung resection, lung transplantation, mediastinal surgery, rib resection, tracheal resection, tracheobronchoplasty, diaphragm plication, esophagectomy, paraesophageal hernia repair

## Abstract

**Simple Summary:**

Robotic surgery was first introduced in the late 1980s. Over the last few decades, it gradually gained popularity across all fields of surgery. Thoracic surgery is one of the surgical subspecialities to embrace this innovative platform. The field is currently experiencing a paradigm shift from the video-assisted thoracoscopic platform to the robotic platform, which will likely become the mainstream approach to minimally invasive surgery. There is still a lack of large randomized control studies to directly compare the patient outcomes between the minimally invasive approaches. This review aims to summarize the current application of the robotic platform in commonly practiced thoracic operations, its impact on clinical outcomes, and the future directions for thoracic robotic surgery to assist surgeons when choosing the optimal surgical approach.

**Abstract:**

With the paradigm shift in minimally invasive surgery from the video-assisted thoracoscopic platform to the robotic platform, thoracic surgeons are applying the new technology through various commonly practiced thoracic surgeries, striving to improve patient outcomes and reduce morbidity and mortality. This review will discuss the updates in lung resections, lung transplantation, mediastinal surgeries with a focus on thymic resection, rib resection, tracheal resection, tracheobronchoplasty, diaphragm plication, esophagectomy, and paraesophageal hernia repair. The transition from open surgery to video-assisted thoracoscopic surgery (VATS) to now robotic video-assisted thoracic surgery (RVATS) allows complex surgeries to be completed through smaller and smaller incisions with better visualization through high-definition images and finer mobilization, accomplishing what might be unresectable before, permitting shorter hospital stay, minimizing healing time, and encompassing broader surgical candidacy. Moreover, better patient outcomes are not only achieved through what the lead surgeon could carry out during surgeries but also through the training of the next generation via accessible live video feedback and recordings. Though larger volume randomized controlled studies are pending to compare the outcomes of VATS to RVATS surgeries, published studies show non-inferiority data from RVATS performances. With progressive enhancement, such as overcoming the lack of haptic feedback, and future incorporation of artificial intelligence (AI), the robotic platform will likely be a cost-effective route once surgeons overcome the initial learning curve.

## 1. Introduction

Robotic surgery has revolutionized thoracic surgery, offering surgeons enhanced dexterity, precision, and visualization, which have collectively elevated the standard of care for patients. The adoption of robotic video-assisted thoracic surgery (RVATS) has allowed for more intricate procedures to be performed minimally invasively, reducing patient trauma, postoperative pain, and hospital stays. Compared to video-assisted thoracoscopic surgery (VATS), robotic systems provide three-dimensional (3D) visualization and articulated instruments, facilitating maneuverability and access to complex anatomical structures. One can argue that the development of 3D-VATS is a comparable option to RVATS as it also provides a high-definition imaging system and utilizes an already existing platform, contributing to wide accessibility and an easier learning curve [1]. However, the results and efficacy of 3D-VATS compared to robotic platforms remains to be studied.

In recent years, robotic surgery for lung pathologies has gained widespread acceptance, driven by evidence of improved patient outcomes, including reduced complications and faster recovery times. The field continues to evolve rapidly, with ongoing technological innovations promising even more refined and efficient procedures.

**Figure 1:**
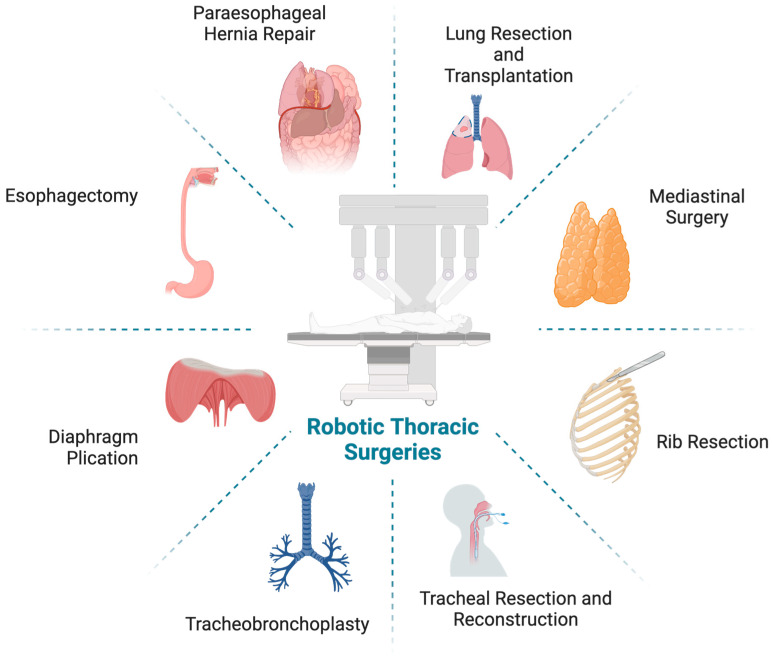
Overview of thoracic surgeries that are performed on the robotic platform (Created with BioRender.com).

Moreover, the robotic platform has become an invaluable educational tool, offering surgical trainees unparalleled exposure to thoracic anatomy and procedures (Figure 1). The integration of dual consoles and recording capabilities allows for a comprehensive learning experience, making RVATS a critical component of modern thoracic surgical training programs [1].

As reflected in recent curricula, structured robotics training is increasingly seen as essential for preparing the next generation of thoracic surgeons to meet the demands of minimally invasive surgery [2].

This review aims to explore the current landscape of robotic thoracic surgery, examining its application across various thoracic procedures, evaluating its impact on patient outcomes, and discussing future directions in this rapidly advancing field.

## 2. Methods

A literature review was conducted between September of 2023 and April 2024 on PubMed for clinical research papers and review papers published after January 2000 for each thoracic surgery discussed in this review paper. Combinations of keywords, including “robotic” and each respective thoracic surgery, were entered in the search bar. Papers that included a combination of keywords in the titles but did not address the pertinent thoracic surgery through the robotic platform were excluded. Each article was assessed, and significant outcomes were extracted to formulate conclusions, summarizing the current application of robotic platforms for commonly practiced thoracic operations. Additional investigations of PubMed relevant to resident training, cost-effectiveness, and artificial intelligence in terms of the adoption of robotic platforms were conducted for the authors to provide a more comprehensive summary.

## 3. Lung Resection

### 3.1. Wedge Resection

Robotic-assisted wedge resection has emerged as a pivotal minimally invasive option for the surgical treatment of lung cancer, the result of the ongoing evolution of the robotic platform. These advancements aim to achieve oncologic outcomes equivalent to traditional approaches while reducing the extent of resection [3]. However, as the adoption of robotic systems becomes more widespread, rigorous studies are needed to assess the feasibility, cost-effectiveness, and long-term outcomes associated with this approach. A significant study by Martin et al. utilized a retrospective propensity match analysis with data from the National Cancer Database, comparing RVATS with traditional VATS in a cohort of 16,559 patients [4]. The study highlighted a statistically significant improvement in lymph node harvesting with RVATS, though it found no significant difference in nodal upstaging between the two approaches. These findings underscore the need for careful consideration of the robotic platform’s cost-effectiveness, operative time, and potential impact on lymph node management before it can be universally adopted as the standard of care.

### 3.2. Segmentectomy

Anatomical segmentectomy, once primarily used for the treatment of bronchiectasis and tuberculosis, has increasingly been adopted for lung cancer resection, thanks to the development of minimally invasive platforms like VATS and RVATS. The robotic platform has proven particularly beneficial for this technically demanding procedure, enabling more precise and accurate resections. Demir et al. were among the first to report comparable morbidity and mortality rates between robotic and non-robotic segmentectomy in their 2014 prospective analysis of 99 patients [5]. While the longer operative time associated with robotic segmentectomy may initially deter some surgeons, the benefits of superior visualization and more complete control over an already challenging operation cannot be overlooked [6]. Subsequent retrospective studies and meta-analyses have consistently demonstrated the safety, feasibility, and oncologic efficacy of robotic segmentectomy [7,8,9,10,11,12,13,14,15,16]. Notably, a comprehensive analysis by Zhang et al. of over 45,000 patients highlighted significant advantages in the robotic group, including reduced blood loss, lower conversion rates, shorter hospital stays, improved lymph node harvest, and better 5-year disease-free survival rates [17]. Other innovations, such as the fissureless technique and the lung-inverted approach described by Yanagiya et al. and Igai et al., further enhance the robotic platform’s utility, allowing surgeons to avoid lung rotation in the confined thoracic cavity while ensuring adequate surgical margins [18,19,20].

### 3.3. Sleeve Lobectomy

Sleeve lobectomy has traditionally been performed through open or hybrid approaches combining VATS and RVATS. However, recent advancements have enabled the complete robotic execution of this procedure, offering significant benefits in terms of precision and patient outcomes. Schmid et al. first demonstrated the feasibility of a fully minimally invasive approach to sleeve lobectomy in 2011, combining VATS with robotic assistance [21]. Building on this foundation, Cerfolio’s 2016 case series reported the successful completion of eight robotic sleeve lobectomies, with only one conversion to open surgery due to bleeding complications [22]. These early successes paved the way for the broader adoption of robotic techniques, with subsequent studies affirming the robotic platform’s applicability to even the most challenging cases involving centrally located tumors [23,24,25,26,27,28,29,30,31,32,33,34]. As the field advances, innovative approaches such as the uniportal hybrid technique explored by Gonzalez-Rivas aim to further reduce the invasiveness of lung resection while maintaining oncologic integrity [35,36]. These developments reflect the growing confidence in robotic surgery as a viable alternative to traditional techniques.

## 4. Lung Transplantation

Lung transplantation, the gold standard treatment for patients with refractory end-stage lung failure, has evolved significantly since the pioneering efforts of Dr. Hardy, Dr. Cooper, and the Toronto Lung Transplant team in the mid- to late-20th century [37]. Traditional surgical approaches, including clamshell incisions, sternotomy, and thoracotomy, while effective, are highly invasive and associated with considerable postoperative morbidity. Patients undergoing these procedures often experience prolonged hospital stays, increased pain, and delayed wound healing, which can lead to a cycle of restricted breathing, narcotic dependence, and potentially lethal complications. The shift towards minimally invasive (MI) techniques in thoracic surgery prompted exploration into their application for lung transplantation. Early attempts, however, faced significant hurdles due to the complexity of the procedure and the challenging anatomy of the thoracic cavity. Despite some reported improvements in postoperative outcomes, including reduced narcotic use and better respiratory function, the propagation of MI lung transplantation remained limited until the advent of robotic-assisted surgery [38,39,40,41,42].

**Figure 2:**
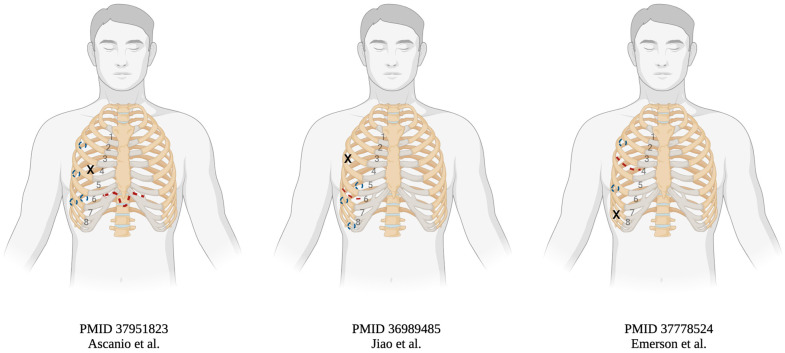
Robotic lung transplantation incision and port placements [44,45,46]. Key: Red dashed lines represent primary incision, blue dashed circles representat port site incisions, blue Xs represent left atrial clamp incision (Created with BioRender.com).

The integration of the robotic platform into lung transplantation marks a significant advancement in the field. One of the primary challenges in robotic lung transplantation is the extraction of the native lung and the introduction of the graft through these minimized incisions. Recent case reports have demonstrated the feasibility of performing lung transplantation through four robotic port incisions and a primary incision as small as 5 to 6 cm, varying in location depending on the surgeon’s preference, displayed in Figure 2 and compiled in Table 1 [43,44,45,46]. For instance, Ascanio et al. have successfully utilized a subxiphoid approach with an 8 cm incision [44]. Jiao et al. prefer an 8 cm incision in the sixth intercostal space while Emerson et al. favor a 6 cm incision in the fourth intercostal space [45,46]. With the precision and dexterity afforded by the robotic arms, surgeons have successfully performed bronchial, venous, and arterial anastomoses. These anastomoses are typically completed using Prolene or Polydioxanone sutures for the bronchial anastomosis, and Gore-Tex or Prolene for the left atrial and pulmonary arterial anastomoses [44,45,46]. Notably, most cases were performed without the need for extracorporeal membrane oxygenation (ECMO) support, although ECMO was utilized in select cases involving high pulmonary pressure or other complications [43]. All case reports reported less postoperative pain as demonstrated by patients being discharged without narcotics.

While these case reports offer promising results, including reduced postoperative pain and the potential for narcotic-free recovery, the data remain limited by small sample sizes and the preliminary nature of the technique. As noted by Jiao et al., the lack of haptic feedback and the necessity of a skilled assistant at the bedside pose challenges to the widespread adoption of this approach [45]. Moreover, Emerson et al. highlighted the technical demands of knot tying and suture management, which require further refinement and technological advancements to ensure broader applicability [46].

As the robotic platform becomes more accessible and the learning curve less steep, especially for younger surgeons trained in these techniques, the potential for robotic lung transplantation to become a standardized practice increases. However, more extensive studies with larger sample sizes and long-term follow-up are needed to validate these early successes and address the current limitations. Currently, robotic lung transplantation represents a safe and feasible option for select patients when performed by an experienced robotic thoracic surgery team. As technology continues to evolve and more data becomes available, the role of robotic surgery in lung transplantation is likely to expand, offering a less invasive alternative with the potential for improved patient outcomes.

## 5. Mediastinal Surgery

Mediastinal surgery encompasses a wide range of pathologies, which are typically classified based on their anatomical location within the anterior, middle, and posterior compartments. The International Thymic Malignancy Interest Group further categorizes these regions into prevascular, visceral, and paravertebral compartments, aligning with the respective anatomical terms. Among these, thymoma remains the most prevalent mediastinal tumor, and consequently, thymectomy has been the focus of extensive research, particularly in refining minimally invasive surgical techniques.

Traditionally, sternotomy has been the gold standard for mediastinal tumor resection, providing optimal exposure but at the cost of significant morbidity. The introduction of minimally invasive surgery in the early 1990s, first with VATS and later with RVATS marked a significant shift towards reducing the invasiveness of these procedures [47,48]. Current research now aims to establish the superiority of these less invasive approaches and to validate their safety and efficacy through long-term outcomes.

Robotic thymectomy is a well-established technique, with numerous studies demonstrating its safety and effectiveness. The dissection during robotic thymectomy has been standardized, typically involving the removal of all tissue encased by the innominate vein superiorly, diaphragm inferiorly, and phrenic nerves laterally. There is an ongoing debate over the optimal surgical approach—whether left-sided, right-sided, or subxiphoid. Proponents of the left-sided approach argue that it offers better visualization of the often variable left phrenic nerve and the frequently overgrown left-sided thymus. Conversely, the right-sided approach is favored for its anatomical landmarks, such as the superior vena cava, larger working space, and reduced risk of trocar injury [49,50]. The subxiphoid approach, initially studied in cadavers and now applied in clinical settings, offers a surgical view akin to sternotomy but with the benefits of minimally invasive access [51,52,53,54]. Wu et al. have taken this a step further, providing preclinical data that supports the feasibility of a single-port subxiphoid approach, which could further reduce the invasiveness of the procedure [55].

Despite the variety of approaches, recent studies have sought to expand the applicability of robotic surgery in mediastinal tumors, challenging traditional exclusion criteria [47,56,57]. Tumor size, for example, has traditionally been a limiting factor; however, studies such as those by Azenha et al. have demonstrated the feasibility of robotic resection for anterior mediastinal tumors up to 9.5 cm in diameter without compromising oncologic outcomes [56]. In another pioneering effort, Tamagawa et al. leveraged the precision of robotic surgery to perform phrenic nerve reconstruction using a segmental intercostal nerve graft in a case of thymoma encasing a segment of the left phrenic nerve [58].

Efforts to further minimize the invasiveness of mediastinal surgery continue to evolve. McCormack et al., for instance, questioned the necessity of chest tube placement following robotic thymectomy. Their study of 75 patients revealed that 74 did not require chest tubes intraoperatively, immediately postoperatively, or within 60 days postoperatively, suggesting that chest tube omission may be safe in select cases [59].

With a wealth of retrospective reviews confirming the safety and feasibility of robotic approaches, there is growing confidence in their application for both benign and malignant mediastinal diseases. However, while several comparative studies have shown equivalence between robotic approaches and sternotomy, the lack of prospective trials limits the ability to definitively claim the superiority of robotic surgery over traditional methods. Future research should focus on addressing these gaps, with larger sample sizes and long-term follow-up to better establish the role of robotics in mediastinal surgery.

## 6. Rib Resection

Rib resection is a cornerstone treatment for thoracic outlet syndrome (TOS), a condition characterized by the compression of neurovascular structures as they pass through the thoracic outlet. The surgical approaches to rib resection are broadly classified into extrathoracic and intrathoracic techniques. Extrathoracic approaches, including posterior, supraclavicular, and transaxillary incisions, have traditionally been used. However, with advancements in surgical technology, intrathoracic approaches have increasingly incorporated minimally invasive techniques, with robotic-assisted surgery emerging as a promising option. Since its first reported use in 2012, the robotic platform has been applied to first rib resection to leverage its associated benefits of reduced morbidity, decreased mortality, and shorter recovery times [60]. One of the most significant advantages of the robotic approach is the ability to eliminate the need for retraction and manipulation of delicate neurovascular structures, thereby reducing the risk of injury.

Kocher et al. published a landmark case series in 2016, demonstrating the feasibility of robotic transthoracic rib resection. In this study, the surgery was initially performed using three robotic ports with an additional 2 cm axillary incision, and later refined to only three robotic ports. The median hospital stay for these patients was 2 days, with short-term postoperative follow-up confirming the resolution of symptoms [61]. These findings were echoed by Gharagozloo et al. in two separate studies. One study included 83 patients with Paget–Schroetter syndrome, and the other included 67 patients with either Paget–Schroetter syndrome or thoracic outlet syndrome [62,63]. Both studies confirmed the feasibility of the robotic approach, with a median hospital stay of three to four days and an intermediate outcome of two years showing 100% subclavian vein patency in patients with vascular thoracic outlet syndrome. Subsequent case series have corroborated these findings, further validating the robotic approach for rib resection [64,65,66,67]. Additionally, case reports of supernumerary rib resections and rib tumor resections have demonstrated the versatility and applicability of the robotic platform in these scenarios [68,69,70].

Efforts to minimize perioperative morbidity have driven further innovations in robotic rib resection techniques. Zehnder et al. explored a complete portal approach using two 8 mm working ports and one 12 mm camera port. Although the operative times reported ranged from 71 to 270 min, reflecting a steep learning curve, the approach demonstrated feasibility [71]. Urena et al. introduced a single-port approach, utilizing three 8 mm robotic trocars through a 4 cm incision in the fifth intercostal space, further refining the technique and reducing invasiveness [72].

A systematic review by Reyes et al. highlighted the advantages of the robotic approach, noting favorable intraoperative and postoperative metrics, such as minimal blood loss, low conversion rates, decreased pain, and low complication rates [73]. Burt et al. provided additional evidence supporting the robotic technique, showing statistically significant reductions in self-reported pain and brachial plexus palsy when compared to the conventional supraclavicular approach [74].

Beyond the immediate surgical outcomes, the robotic approach to rib resection offers significant educational benefits. As emphasized by Lazzaro, the superior visualization of critical neurovascular structures and the standardized 12-step robotic approach have transformed the learning curve for this procedure. The enhanced visualization and precise instrumentation provided by the robotic platform allow mentors to better teach the nuances of this complex surgery, potentially reducing surgical morbidity in future generations of thoracic surgeons [75,76,77,78]. While the current data are promising, further research is needed to refine these techniques and assess long-term outcomes. As robotic technology continues to evolve, there is potential for even greater improvements in the safety, efficacy, and educational value of robotic-assisted rib resection.

## 7. Tracheal Resection and Reconstruction

Tracheal resection and reconstruction are among the most challenging procedures in thoracic surgery, largely due to the rarity of primary tracheal tumors and the anatomical complexity involved. Primary tracheal tumors, including leiomyoma, leiomyosarcoma, squamous cell carcinoma, and adenoid cystic carcinoma, require precise surgical management to ensure complete resection and minimize the risk of recurrence. Historically, open surgical approaches have been the gold standard for these resections, given the limitations of bronchoscopic and local excisions. However, as with other thoracic procedures, tracheal surgery has progressively transitioned from open to minimally invasive approaches, including video-assisted and now robotic-assisted techniques.

The application of robotic surgery to tracheal resection was first demonstrated by Jiao et al. in 2015, who successfully performed a non-circumferential tracheal resection and anastomosis for leiomyoma [79]. The procedure utilized three robotic ports placed in the seventh intercostal space (posterior midaxillary line and posterior axillary line) and the fifth intercostal space (anterior axillary line), with an additional assist incision through the third intercostal space. A partial lateral wall resection of a 2.5 cm × 2 cm area was performed, followed by a running anastomosis with 2-0 Prolene sutures.

Building on this early success, Qiu et al. in 2018 reported the first circumferential tracheal resection and reconstruction for a primary extra-luminal tracheal leiomyosarcoma located above the carina [80]. This procedure was conducted entirely through a portal approach utilizing four robotic ports. Due to a size mismatch between the proximal and distal ends of the trachea post-resection, the anastomosis was performed in a telescope fashion, with the smaller proximal end pulled into the larger distal end, secured by a 2-0 polypropylene continuous running suture. This case exemplifies the adaptability of the robotic platform in addressing complex anatomical challenges.

In addition to these technical advancements, there have been innovations in anesthetic management during robotic tracheal surgery. In 2021, Li et al. introduced a non-intubated approach using a laryngeal mask airway without muscle relaxants in a small cohort of five carefully selected patients [81]. While this technique offers potential benefits in reducing airway trauma and anesthesia-related complications, it also raises significant concerns. As noted by Zalepugas et al., the lack of a secured airway increases the risk of uncontrolled bleeding, and the absence of muscle relaxants poses challenges in managing intraoperative coughing. Additionally, the use of electrosurgery in a high-oxygen environment elevates the risk of airway fires. Although initial outcomes are promising, larger studies are necessary to evaluate the safety and broader applicability of this approach [82].

Further expanding the scope of robotic tracheal surgery, Spaggiari et al. in 2023 successfully resected an adenoid cystic carcinoma using extracorporeal membrane oxygenation (ECMO) support, bypassing the need for intubation [83]. This approach provided excellent surgical exposure and ensured adequate respiratory support, although it also introduced risks such as renal failure, bacterial pneumonia, bleeding, and hemolysis, which are inherent to ECMO-assisted procedures. The successful application of ECMO in this context underscores the versatility of the robotic platform, yet also highlights the need for careful patient selection and management of potential complications.

Overall, robotic-assisted tracheal resection and reconstruction offer significant advantages in terms of dexterity, precision, and visualization. The ability to perform intricate suturing, maintain high-dimensional views, and carefully manage vascular structures makes the robotic platform an invaluable tool in these complex procedures. However, the adoption of these techniques requires not only advanced surgical skills but also further technological refinement and robust clinical evidence to fully establish their role in thoracic surgery.

## 8. Tracheobronchoplasty

Tracheobronchoplasty is a surgical procedure designed to treat tracheobronchomalacia, a chronic condition characterized by the loss of the normal D-shape configuration of the trachea and mainstem bronchi, leading to excessive compliance of the pars membranacea. This condition results in the narrowing and potential collapse of the central airways, particularly during instances of increased intrathoracic pressure. The incidence of tracheobronchomalacia varies widely, reported between 1% and 13%, largely due to its complex presentation, which often mimics other pulmonary diseases [84].

The etiology of tracheobronchomalacia can be either primary (congenital) or secondary (acquired). Diagnosis typically relies on dynamic imaging techniques, such as computerized tomography (CT) and bronchoscopy, to visualize airway collapse, which must exceed 70% to confirm the condition [85]. Severity is graded as mild (70–80% collapse), moderate (81–90%), and severe (>90%).

Despite advances in medical technology, the surgical management of tracheobronchomalacia has remained relatively stagnant, with tracheobronchoplasty traditionally performed through a right posterolateral thoracotomy, as first described by Nissen in 1954 [86]. Although VATS has been applied to various lung pathologies, including tracheobronchomalacia [87], the limitations of rigid instrumentation and 2-dimensional (2D) visualization have prevented a significant shift from open surgery to minimally invasive approaches. This reliance on open thoracotomy has limited the availability of tracheobronchoplasty to patients who might otherwise benefit from less invasive techniques.

The advent of robotic-assisted surgery has introduced new possibilities for tracheobronchoplasty. Lazzaro et al. pioneered a minimally invasive approach using the robotic platform, attributing their success to the enhanced dexterity provided by the robotic arms and the superior 3D visualization [88,89]. This approach was validated in a subsequent case series involving 42 patients, which demonstrated low morbidity and mortality associated with the robotic technique. However, as Milman and Ng critically noted, the lack of long-term data and the absence of clearly defined patient selection criteria suggest that conclusions about the safety and efficacy of robotic tracheobronchoplasty may be premature [90]. Follow-up studies on the original cohort of 42 patients provided some intermediate-term data, showing a significant increase in postoperative forced expiratory volume in 1 s (FEV1) at a median follow-up of 5 months, sustained through a median of 30 months. Additionally, there was a 0% 30- and 90-day mortality rate, and patients reported a significant decrease in total St. George’s Respiratory Questionnaire (SGRQ) impact scores, indicating improved quality of life [91]. Seastedt et al. corroborated these findings in a single-center study, suggesting that minimally invasive approaches could be inclusive of a broader range of tracheobronchomalacia patients [92].

An important aspect of robotic tracheobronchoplasty that remains under investigation is patient selection. In a study by Inra et al., a cohort of 108 patients undergoing robotic-assisted tracheobronchoplasty was stratified by the severity of airway obstruction using the Global Initiative for Obstructive Lung Disease (GOLD) classification system [93]. The study found that patients classified as GOLD 2 and 3 experienced statistically significant improvements in FEV1, while the sample size for those with the most severe disease (GOLD 4) was too small to draw definitive conclusions. This study, the largest case series to date, underscores the need for larger cohorts and long-term follow-up to better understand which patients are most likely to benefit from robotic tracheobronchoplasty.

The procedural standardization of robotic tracheobronchoplasty, as laid out by Lazzaro et al., provides a clear framework for performing this complex surgery [89]. However, specific cases demonstrate the need for flexibility in approach, such as using an EZ blocker instead of double-lumen tubes to overcome technical challenges [94]. While the robotic approach shows promise, ongoing research is essential to refine patient selection criteria, optimize surgical techniques, and gather more robust long-term data.

## 9. Diaphragm Plication

Diaphragm plication is a surgical intervention designed to address diaphragm eventration, a condition characterized by abnormal elevation of part or the entire hemidiaphragm. This condition can arise from both primary congenital etiologies and secondary acquired causes, such as trauma or tumors affecting the phrenic nerve. The resulting diaphragmatic dysfunction can lead to a range of symptoms, including respiratory issues like dyspnea, orthopnea, and recurrent respiratory infections, as well as gastrointestinal symptoms due to increased intraabdominal pressure, such as dyspepsia, dysphagia, and gastroesophageal reflux.

Historically, diaphragm plication was performed using an open transthoracic approach, first described in 1923 by Morison. This method, typically involving a posterolateral thoracotomy through the sixth, seventh, or eighth intercostal space, was the standard treatment for symptomatic patients [95]. However, the advent of minimally invasive surgery marked a significant shift in the approach to diaphragm plication, offering advantages such as reduced postoperative pain, shorter hospital stays, and improved quality of life and prompting surgeons to adopt these methods more widely [96,97,98].

The diaphragm can be accessed either thoracoscopically or laparoscopically, leading to a debate over the optimal approach. The introduction of the robotic platform has further fueled this discussion, offering enhanced precision and control. In 2012, Kwak et al. reported the first case of robotic-assisted thoracoscopic diaphragm plication, demonstrating the feasibility of this approach [99]. Subsequent studies, including those by Ahn et al., have explored the simultaneous use of laparoscopy with the robotic platform to mitigate the lack of tactile feedback and minimize the risk of injury [100]. These studies, along with various case reports, have established the safety and effectiveness of robotic-assisted diaphragm plication in a range of scenarios, including traumatic diaphragmatic hernias and diaphragm paralysis secondary to procedures like cryoballoon ablation or conditions such as COVID-19 [101,102,103,104,105]. One of the key advantages of the robotic platform is its ergonomic design, which allows for more precise suturing, particularly near challenging anatomical areas like the costophrenic and cardiophrenic angles. Xu et al. demonstrated this benefit in pediatric populations, showing a significant reduction in suturing time with the robotic approach. Although the overall operative time for robotic-assisted plication may be comparable to conventional thoracoscopic techniques due to the time required for robotic setup, the precision and control offered by the robotic arms are significant advantages [106]. Additionally, Lampridis et al. extrapolated a reduction in operative time and blood loss when comparing to that of robotic transabdominal approaches, highlighting the potential benefits of this minimally invasive technique [107,108].

Despite these advantages, there are drawbacks associated with the transthoracic approach, particularly the limited workspace imposed by the ribcage and the eventration itself. This has led some surgeons to advocate for the transabdominal approach, which offers access to both sides of the diaphragm, potentially causes less postoperative pain from abdominal ports compared to intercostal ports, decreases the risk of injury to abdominal viscera and lungs, and eliminates the need for selective ventilation [109,110]. However, as Du et al. emphasized, the choice between transthoracic and transabdominal approaches should be guided by careful patient selection and a personalized, multidisciplinary approach to optimize outcomes.

Retrospective studies and review papers have sought to differentiate the outcomes between open and minimally invasive approaches, as well as between transthoracic and transabdominal techniques for diaphragm plication [108,111]. However, current analyses are limited by small sample sizes, single-center retrospective designs, and a lack of long-term outcome data. These limitations underscore the need for large prospective trials to definitively establish the superiority of one technique over the others.

## 10. Esophagectomy

Esophagectomy is a complex surgical procedure often required for the treatment of esophageal malignancies and certain benign conditions. Given the intricate anatomy and critical functions of the esophagus, these surgeries are associated with significant risks and challenges. Traditionally, esophagectomies were performed using open surgical techniques, which, although effective, resulted in considerable morbidity and prolonged recovery periods. In recent years, the field of esophageal surgery has undergone a paradigm shift with the introduction of minimally invasive techniques, particularly robotic-assisted esophagectomy, which has emerged as a promising alternative. Robotic-assisted esophagectomy offers several advantages over traditional open surgery and even conventional minimally invasive approaches, such as VATS. The robotic platform provides enhanced precision through articulated instruments, superior three-dimensional visualization, and improved ergonomics for the surgeon. These benefits have contributed to the growing adoption of robotic esophagectomy since its introduction in the early 2000s [112,113,114].

Several randomized controlled trials and meta-analyses have directly compared robotic-assisted minimally invasive esophagectomy (RAMIE) with other surgical approaches, providing valuable insights into its efficacy and safety. For example, Watanabe et al. conducted a comprehensive meta-analysis that highlighted several advantages of the robotic approach. These included notably fewer pulmonary complications, more accurate lymphadenectomy, and comparable or reduced blood loss, operative time, and overall complication rates. Importantly, no significant differences were observed in key postoperative metrics, such as hospital stay length, ICU stay, and 30- and 90-day mortality rates [115,116,117,118].

However, despite these promising results, robotic-assisted esophagectomy is not without challenges. One of the most concerning issues is the observed higher rate of anastomotic leaks and the need for reoperation compared to other techniques. This complication is likely related to the steep learning curve associated with performing precise intrathoracic anastomoses using the robotic platform. As surgeons gain more experience and refine their techniques, it is anticipated that these outcomes will improve [119,120].

In addition to surgical outcomes, the cost-effectiveness of robotic-assisted esophagectomy has been a topic of considerable interest. While the initial costs associated with the robotic platform are higher, studies have shown that these expenses may be offset by the reduced incidence of complications, such as pulmonary issues, and shorter hospital stays in some cases. This has led to a more balanced view of the financial implications of robotic esophagectomy, suggesting that it may not impose an additional financial burden compared to traditional methods when considering the overall cost of care [121].

## 11. Paraesophageal Hernia Repair

The adoption of a robotic platform to carry out paraesophageal hernia (PEH) repair expanded in the early 2000s, including application to rare congenital cases and uncommon hernia pathologies [122,123,124]. The robotic platform offers several advantages over traditional laparoscopic approaches, including enhanced three-dimensional visualization, improved dexterity through articulated instruments, and superior ergonomics for the surgeon. These benefits are especially pertinent given the complexity of PEH repair, which often requires meticulous reduction in a large hernia sac, extensive esophageal mobilization, and precise crural closure to prevent recurrence.

Gehrig et al. were one of the first studies that compared robotic-assisted platforms to those of conventional laparoscopic and open approaches [125]. With a database of 42 patients, though the operative time was found to be 38 min longer utilizing the robotic approach, the postoperative complication and hospital stay were less compared to open and comparable to the laparoscopic approach, showing that the robotic approach is a non-inferior option to that of laparoscopic. Galvani et al. and several other groups then reached similar conclusions either through a more expanded sample size or demonstrated applicability in different populations [126,127,128]. Additionally, Sarkaria et al. showed that with mastery of laparoscopic techniques as do most of the new generation of surgeons, the learning curve to adopt the robotic technique is relatively short [129].

Over the past two years, more data have been published to provide a direct comparison between the laparoscopic and robotic approaches to paraesophageal hernia. Bhatt and Wei completed a systemic review of the perioperative outcomes and found that the robotic approach has an overall decreased conversion rate and shorter hospital stay compared to that of the laparoscopic approach. However, one large study of 17,000 patients had an association with a higher rate of esophageal perforation, which might be explained by the lack of tactile feedback on the robotic platform [130]. Another limitation of the robotic platform that was mentioned was the cost-effectiveness of the approach and its applicability to economically limited areas. Lekarcyzk et al. concluded that although the robotic repairs were associated with higher supply costs and charges, the hospital profits were similar when compared to that of laparoscopic hernia surgeries [131]. Additionally, with experienced surgeons and the shorter learning curve, Prasath et al. suspected that the robotic platform would provide long-term outcomes to make its applicability cost-effective [132].

In addition, with the dexterity and better visualization provided by the robot, cases of recurrent hernias proved to have harder dissection, increased peri-operative complication rates, and conversion outcomes were shown to have benefited from the robotic platform [133,134,135,136]. To take another step forward, the Belsey Mark IV operation was also carried out robotically as demonstrated by Reza et al. [137]. In patients with a hostile abdomen, the Belsey Mark IV operation is offered to approach the paraesophageal hernia transthoracically. Reza et al. published a case series of five patients who underwent robotic Belsey Mark IV operation, demonstrating an average operative time of 209 min, postoperative length of stay of 4.2 days and an average blood loss of 67 mL [137].

## 12. Conclusions

This review has highlighted the significant advancements in robotic surgery across a range of thoracic pathologies, demonstrating its potential to enhance surgical precision, reduce patient morbidity, and improve overall outcomes. While the robotic platform has proven to be particularly beneficial in technically challenging procedures such as rib resection and tracheal reconstruction, it also offers a unique advantage in surgical education. The enhanced visualization and dexterity provided by the robotic system likely facilitate the teaching and transfer of complex surgical techniques to the next generation of surgeons, despite the associated learning curve, as there is no limitation to the trainee’s visual learning if the trainee has access to either real-time platform or recording.

One of the most significant limitations of robotic surgery has been the lack of haptic feedback, a challenge that may soon be mitigated with the introduction of the Da Vinci 5 surgical system by Intuitive, which offers the option to toggle force feedback on and off, delivering less force on tissue and providing the sensation of push and pull forces through instrument tips. This innovation, along with improved ergonomics designed to extend the careers of surgeons, represents a crucial step forward in addressing the physical demands of robotic surgery. Moreover, as the field of surgical data science evolves, more operative data will be available to surgeons for further improvements in technique, and further expanding the capabilities of robotic systems. Looking ahead, the integration of artificial intelligence (AI) into thoracic surgery marks a new beginning rather than an endpoint in the evolution of robotic platforms. As these technologies continue to develop, they will likely address the current limitations of robotic surgery and open new avenues for innovation in thoracic procedures.

As most studies, including this this review, assess the efficacy of the robotic platform mostly through quantifiable intraoperative and postoperative outcomes, one issue that lacks attention is the management of intraoperative catastrophes, which has been reported to range from 0.5 to 2.6% [138]. The robotic platform and telesurgery require the surgeon to be away from the operating table at the console. Therefore, careful coordination among all individuals of the surgical team and prior development of contingency plans are needed [139]. Most intraoperative catastrophes in thoracic surgical cases are likely vascular events, and the role of compression with a rolled gauze is one of the listed and crucial items in emergency plans. Several advantages of the robotic platform come to mind: the constant presence of rolled gauze inside the thoracic cavity, the immediate presence of a robotic grasper for compression, the stability of compression provided by the robotic arms, and the ability to verify hemostasis in the thoracic cavity after conversion but pending on degree of docking. At our institution, a hemostatic and topical agent, EVARREST patch (Ethicon, Inc., Somerville, NJ, USA) is part of our contingency plan and can be applied robotically to allow us to remain minimally invasive and address pulmonary arterial bleeding, especially if bleeding is from an arterial branch that will be transected. However, we do have a low threshold to convert to open to obtain proximal and distal control. A sponge stick is up on the field for every robotic case in preparation for a pulmonary arterial bleed that is not able to be controlled with robotic arms. The assistant is responsible for inserting the sponge stick through the assist port and tamponade the bleeding while the robot is unlocked to perform an emergent thoracotomy. As Sakakura et al. demonstrated, emergency contingency plans and rollout need to be carefully developed and repeatedly practiced, allowing for smooth coordination and execution [139].

Additionally, cost-effectiveness remains a frequently debated issue in the adoption of robotic surgery within thoracic practice. However, as several institutional analyses have shown, the initially higher costs associated with the robotic platform—largely due to the learning curve and longer operative times—can be mitigated over time. As surgeons overcome the learning curve and institutions increase their case volumes, the economic impact of robotic surgery becomes more favorable, making it a viable option in thoracic surgery [140,141,142,143].

In conclusion, while larger volume studies and long-term outcome research are necessary to further validate the safety and efficacy of robotic surgery in thoracic applications, the ongoing technological advancements suggest a promising future. The adoption of the robotic platform, coupled with emerging AI applications, is poised to transform thoracic surgery, enhancing both the quality of patient care and the surgical profession itself [144].

## Figures and Tables

**Table 1:** Compilation of published robotic-assisted lung transplantation cases [44,45,46].

Authors (Years)	Demographic (Age/Gender)	Indication	Transplanted Side	Approach	Implant Time (min)	On Pump (Y/N)	Postoperative Course
Ascanio et al. (2023)	65 M	Interstitial pneumonia	Not specified	8 cm subxiphoid and 4 ports	N/A	N	Regular analgesia, no opioids
	71 M	IPF	Left	as above	N/A	N	Regular analgesia, no opioids
Jiao et al. (2023)	59 M	COPD	Right	8 cm 6th intercostal incision and 4 ports	WIT 72	N	Discharged POD19
Emerson et al. (2024)	69 M	COPD	Right	6 cm 6th intercostal incision, 8th intercostal incision, 2 ports	WIT 88 ^a^	N	Discharged POD11, no opioids
	63 M	IPF	Right	as above	WIT 111	Y	N/A
	73 F	COPD	Right	as above	WIT 85 ^a^	N	N/A
	67 M	COPD	Bilateral	as above	WIT 68/86	N/N	N/A
	71 F	COPD	Bilateral ^b^	as above	WIT 81/NA	N/Y	N/A
	66 M	COPD	Right	as above	WIT 58	N	N/A
	64 F	IPF	Bilateral	as above	WIT 67/78 ^a^	N/N	N/A
	66 M	IPF	Right	as above	WIT 66	Y	N/A

COPD: chronic obstructive pulmonary disease; F: female; IPF: idiopathic pulmonary fibrosis; M: male; N: no; N/A: not available; POD: post-operative day; WIT: warm ischemia time; Y: yes. ^a^ Robotic assistance to bronchial and left atrial anastomoses only [43]. ^b^ Robotic assistance to right side only [43].

## Data Availability

No new data were created or analyzed in this study. Data sharing is not applicable to this article.

## References

[1] Linsky P.L., Wei B. (2018). Training in robotic thoracic surgery. J. Vis. Surg..

[2] Raad W.N., Ayub A., Huang C.-Y., Guntman L., Rehmani S.S., Bhora F.Y. (2018). Robotic Thoracic Surgery Training for Residency Programs: A Position Paper for an Educational Curriculum. Innov. Technol. Tech. Cardiothorac. Vasc. Surg..

[3] Altorki N., Wang X., Kozono D., Watt C., Landrenau R., Wigle D., Port J., Jones D.R., Conti M., Ashrafi A.S. (2023). Lobar or Sublobar Resection for Peripheral Stage IA Non–Small-Cell Lung Cancer. N. Engl. J. Med..

[4] Martin J.L., Mack S.J., Rshaidat H., Collins M.L., Whitehorn G.L., Grenda T.R., Evans N.R., Okusanya O.T. (2024). Wedge Resection Outcomes: A Comparison of Video-Assisted and Robot-Assisted Wedge Resections. Ann. Thorac. Surg..

[5] Demir A., Ayalp K., Ozkan B., Kaba E., Toker A. (2015). Robotic and video-assisted thoracic surgery lung segmentectomy for malignant and benign lesions. Interact. Cardiovasc. Thorac. Surg..

[6] Baste J.M., Soldea V., Lachkar S., Rinieri P., Sarsam M., Bottet B., Peillon C. (2018). Development of a precision multimodal surgical navigation system for lung robotic segmentectomy. J. Thorac. Dis..

[7] Liang H., Liang W., Zhao L., Chen D., Zhang J., Zhang Y., Tang S., He J. (2018). Robotic versus Video-assisted Lobectomy/Segmentectomy for Lung Cancer: A Meta-analysis. Ann. Surg..

[8] Ferrari-Light D., Cerfolio R.J. (2019). Non-small cell lung cancer 2 cm or less: Robotic segmentectomy sets the gold standard against non-surgical therapy. Ann. Transl. Med..

[9] Nguyen D., Gharagozloo F., Tempesta B., Meyer M., Gruessner A. (2019). Long-term results of robotic anatomical segmentectomy for early-stage non-small-cell lung cancer. Eur. J. Cardio-Thorac. Surg..

[10] Xie B., Sui T., Qin Y., Miao S., Jiao W. (2019). [Comparison of Short-term Outcomes of Lung Segmentectomy by Robotic-assisted and Video-assisted Thoracoscopic Surgery]. Zhongguo Fei Ai Za Zhi.

[11] Kagimoto A., Tsutani Y., Izaki Y., Handa Y., Mimae T., Miyata Y., Okada M. (2020). Initial experience of robotic anatomical segmentectomy for non-small cell lung cancer. Ultrasound Med. Biol..

[12] Zhang Y., Chen C., Hu J., Han Y., Huang M., Xiang J., Li H. (2020). Early outcomes of robotic versus thoracoscopic segmentectomy for early-stage lung cancer: A multi-institutional propensity score-matched analysis. J. Thorac. Cardiovasc. Surg..

[13] Zhou Q., Huang J., Pan F., Li J., Liu Y., Hou Y., Song W., Luo Q. (2020). Operative outcomes and long-term survival of robotic-assisted segmentectomy for stage IA lung cancer compared with video-assisted thoracoscopic segmentectomy. Transl. Lung Cancer Res..

[14] Gergen A.K., White A.M., Mitchell J.D., Meguid R.A., Fullerton D.A., Scott C.D., Weyant M.J. (2021). Introduction of robotic surgery leads to increased rate of segmentectomy in patients with lung cancer. J. Thorac. Dis..

[15] Kodia K., Razi S.S., Alnajar A., Nguyen D.M., Villamizar N. (2021). Comparative Analysis of Robotic Segmentectomy for Non-Small Cell Lung Cancer: A National Cancer Database Study. Innov. Technol. Tech. Cardiothorac. Vasc. Surg..

[16] Mao J., Tang Z., Mi Y., Xu H., Li K., Liang Y., Wang N., Wang L. (2021). Robotic and video-assisted lobectomy/segmentectomy for non-small cell lung cancer have similar perioperative outcomes: A systematic review and meta-analysis. Transl. Cancer Res..

[17] Zhang J., Feng Q., Huang Y., Ouyang L., Luo F. (2022). Updated Evaluation of Robotic- and Video-Assisted Thoracoscopic Lobectomy or Segmentectomy for Lung Cancer: A Systematic Review and Meta-Analysis. Front. Oncol..

[18] Yanagiya M., Nagano M., Nakajima J. (2023). Fissureless technique of robotic left lingular segmentectomy for primary lung cancer with incomplete fissure: A case report. J. Cardiothorac. Surg..

[19] Igai H., Nii K., Kamiyoshihara M. (2024). Robotic upper division segmentectomy of the left upper lobe without turning the lung. Multimed. Man. Cardio-Thorac. Surg..

[20] Igai H., Nii K., Kamiyoshihara M. (2024). Two cases of lower lobe segmentectomy (left and right) using the lung-inverted approach in a robotic operation. Multimed. Man. Cardio-Thorac. Surg..

[21] Schmid T., Augustin F., Kainz G., Pratschke J., Bodner J. (2011). Hybrid Video-Assisted Thoracic Surgery-Robotic Minimally Invasive Right Upper Lobe Sleeve Lobectomy. Ann. Thorac. Surg..

[22] Cerfolio R.J. (2016). Robotic sleeve lobectomy: Technical details and early results. J. Thorac. Dis..

[23] Lin M.-W., Kuo S.-W., Yang S.-M., Lee J.-M. (2016). Robotic-assisted thoracoscopic sleeve lobectomy for locally advanced lung cancer. J. Thorac. Dis..

[24] Zhao Y., Chen H., Qiu T., Xuan Y., Luo Y., Shen Y., Jiao W. (2016). Robotic-assisted sleeve lobectomy for right upper lobe combining with middle lobe resection of lung cancer. J. Vis. Surg..

[25] Jo M.S., Kim D.Y., Jeong J.Y., Lee G.D. (2017). Robotic sleeve lobectomy with four arms for lung cancer centrally located in the right lower lobe: A case report. J. Cardiothorac. Surg..

[26] Qiu T., Zhao Y., Xuan Y., Jiao W. (2017). Robotic-assisted double-sleeve lobectomy. J. Thorac. Dis..

[27] Tan G.J.S., Poon J.S., Khoo P.L.Z., Yoong A.W.H., Nardini M., Dunning J. (2018). Robotic left lower sleeve lobectomy with bronchoplasty for the removal of a carcinoid tumour. J. Vis. Surg..

[28] Durand M. (2019). Four-arm robotic sleeve right upper lobectomy. Ann. Cardiothorac. Surg..

[29] Egberts J.H., Moller T., Becker T. (2019). Robotic-Assisted Sleeve Lobectomy Using the Four-Arm Technique in the DaVinci Si(R) and Xi(R) Systems. Thorac. Cardiovasc. Surg.

[30] Jiao W., Zhao Y., Qiu T., Xuan Y., Sun X., Qin Y., Liu A., Sui T., Cui J. (2019). Robotic Bronchial Sleeve Lobectomy for Central Lung Tumors: Technique and Outcome. Ann. Thorac. Surg..

[31] Qiu T., Zhao Y., Xuan Y., Qin Y., Niu Z., Shen Y., Jiao W. (2020). Robotic sleeve lobectomy for centrally located non–small cell lung cancer: A propensity score–weighted comparison with thoracoscopic and open surgery. J. Thorac. Cardiovasc. Surg..

[32] Watkins A.A., Quadri S.M., Servais E.L. (2021). Robotic-Assisted Complex Pulmonary Resection: Sleeve Lobectomy for Cancer. Innov. Technol. Tech. Cardiothorac. Vasc. Surg..

[33] Paglialunga P.L., Molins L., Guzman R., Guirao A., Grando L., Sanchez-Lorente D., Guerrero C., Bello I., Quiroga N., Boada M. (2023). Starting a robotic thoracic surgery program: From wedge resection to sleeve lobectomy in six months. Initial conclusions. Cir. Esp. (Engl. Ed.).

[34] Jacob A., Stamenkovic S.A. (2024). Robotic-assisted thoracic surgery: Left upper lobe sleeve lobectomy for an endobronchial tumour. Multimed. Man. Cardio-Thorac. Surg..

[35] Gonzalez-Rivas D., Koziej P.-H., Sediqi S., Ruprecht B., Jostmeyer H., Valdivia D. (2023). Uniportal hybrid robotic-assisted right upper sleeve lobectomy in an 83-year-old patient with severe pulmonary hypertension. Ann. Cardiothorac. Surg..

[36] Gonzalez-Rivas D., Prado R.F., Garcia-Perez A., Bosinceanu M.L., Motas N., Manolache V. (2023). Bilateral uniportal robotic-assisted thoracic surgery (RATS) sleeve lobectomy for a bilateral endobronchial lung cancer. Ann. Cardiothorac. Surg..

[37] Toronto Lung Transplant G. (1986). Unilateral lung transplantation for pulmonary fibrosis. N. Engl. J. Med..

[38] DeVito Dabbs A.D., Dew M., Zaldonis D., Aubrecht J., Crespo M., Pilewski J., Bhama J., Gilbert S., Bermudez C., Toyoda Y. (2010). 82: Patient-Reported Outcomes after the Minimally Invasive Approach to Lung Transplantation. J. Heart Lung Transplant..

[39] Ahmed H., Zeschky C., Alayyar M., Husain M., Jothidasan A., Padukone A., Bello S., Marczin N., Smail H., Stock U. (2022). Long Term Outcomes of Minimally Invasive Lung Transplantation Compared to Clamshell Approach. J. Heart Lung Transplant..

[40] Thomas J., Chen Q., Malas J., Barnes D., Roach A., Peiris A., Premananthan S., Krishnan A., Rowe G., Gill G. (2024). Impact of minimally invasive lung transplantation on early outcomes and analgesia use: A matched cohort study. J. Heart Lung Transplant..

[41] Fischer S., Strüber M., Simon A.R., Anssar M., Wilhelmi M., Leyh R.G., Harringer W., Haverich A. (2001). Video-assisted minimally invasive approach in clinical bilateral lung transplantation. J. Thorac. Cardiovasc. Surg..

[42] Marczin N., Popov A.F., Zych B., Romano R., Kiss R., Sabashnikov A., Soresi S., De Robertis F., Bahrami T., Amrani M. (2016). Outcomes of minimally invasive lung transplantation in a single centre: The routine approach for the future or do we still need clamshell incision?. Interact. Cardiovasc. Thorac. Surg..

[43] Ascanio F., Royo-Crespo I., Rosado J., Sánchez L., Romero L., Durán-Rey D., Sánchez-Margallo F., Jauregui A. (2023). Advances in robotic lung transplantation: Development and validation of a new surgical technique in animal models. Interdiscip. Cardiovasc. Thorac. Surg..

[44] Ascanio F., Royo-Crespo I., Jauregui A. (2023). Robotic Lung Transplantation: A Paradigm Shift in Surgical Strategy. Arch. Bronc..

[45] Jiao W., Yang R., Zhao Y., Ge N., Qiu T., Sun X., Liu Y., Li K., Li Z., Yu W. (2023). Robot-assisted single lung transplantation. Chin. Med. J. (Engl.).

[46] Emerson D.C.P., Rampolla R., Chikwe J., Megna D. (2024). Robotic-assisted lung transplantation: First in man. J. Heart Lung Transplant..

[47] Burt B.M., Yao X., Shrager J., Antonicelli A., Padda S., Reiss J., Wakelee H., Su S., Huang J., Scott W. (2017). Determinants of Complete Resection of Thymoma by Minimally Invasive and Open Thymectomy: Analysis of an International Registry. J. Thorac. Oncol..

[48] Wu W.-J., Zhang F.-Y., Xiao Q., Li X.-K. (2021). Does robotic-assisted thymectomy have advantages over video-assisted thymectomy in short-term outcomes? A systematic view and meta-analysis. Interact. Cardiovasc. Thorac. Surg..

[49] Coco D., Leanza S. (2023). Robotic thymectomy: A review of techniques and results. Pol. J. Cardio-Thorac. Surg..

[50] Su K.W., Luketich J.D., Sarkaria I.S. (2022). Robotic-assisted minimally invasive thymectomy for myasthenia gravis with thymoma. JTCVS Tech..

[51] Grigoroiu M., Rheinwald M., Ryckembusch L., Kemper J., Brian E., Boddaert G., Seguin-Givelet A., Mariolo A.V. (2022). Full subcostal subxiphoid robotic-assisted radical thymectomy: Preclinical cadaveric study for optimizing patient positioning, table settings, and port configuration. Updat. Surg..

[52] Shimomura M., Ishihara S., Okada S., Inoue M. (2022). Robotic subxiphoid-optical thymectomy. Interact. Cardiovasc. Thorac. Surg..

[53] Zhang H., Wang F., Qiu G., Li Z., Chen L.-Q., Wang Y. (2022). Surgical Tips to Improve Completeness of Transsubxiphoid Robotic Extended Thymectomy. Ann. Thorac. Surg..

[54] E H., Yang C., Zhang L., Xia L., Xu L., Song N., Hu X., Zhu Y., Chen C., Zhao D. (2023). Perioperative outcomes comparison of robotic and video-assisted thoracoscopic thymectomy for thymic epithelial tumor: A single-center experience. Updat. Surg..

[55] Wu C.F., Cheng C., Suen K.H., Stein H., Chao Y.K. (2023). A Preclinical Feasibility Study of Single-Port Robotic Subcostal Anatomical Lung Resection and Subxiphoid Thymectomy Using the da Vinci((R)) SP System. Diagnostics.

[56] Azenha L.F., Deckarm R., Minervini F., Dorn P., Lutz J., Kocher G.J. (2021). Robotic vs. Transsternal Thymectomy: A Single Center Experience over 10 Years. J. Clin. Med..

[57] Geraci T.C., Ferrari-Light D., Pozzi N., Cerfolio R.J. (2021). Midterm Results for Robotic Thymectomy for Malignant Disease. Ann. Thorac. Surg..

[58] Tamagawa S., Hashimoto K., Ichinose J., Matsuura Y., Nakao M., Okumura S., Satoh Y., Mun M. (2023). Phrenic nerve interposition in a completely portal robotic thymectomy. JTCVS Tech..

[59] McCormack A.J., El Zaeedi M., Dorsey M., Cerfolio R.J. (2023). A chest tube after robotic thymectomy is unnecessary. JTCVS Open.

[60] Gharagozloo F., Meyer M., Tempesta B.J., Margolis M., Strother E.T., Tummala S. (2012). Robotic en bloc first-rib resection for Paget-Schroetter disease, a form of thoracic outlet syndrome: Technique and initial results. Innovations.

[61] Kocher G.J., Zehnder A., Lutz J.A., Schmidli J., Schmid R.A. (2018). First Rib Resection for Thoracic Outlet Syndrome: The Robotic Approach. World J. Surg..

[62] Gharagozloo F., Meyer M., Tempesta B., Gruessner S. (2019). Robotic transthoracic first-rib resection for Paget–Schroetter syndrome. Eur. J. Cardio-Thorac. Surg..

[63] Gharagozloo F., Meyer M., Tempesta B., Werden S. (2020). Robotic First Rib Resection for Thoracic Outlet Syndrome. Surg. Technol. Int..

[64] Pupovac S.S., Lee P.C., Zeltsman D., Jurado J., Hyman K., Singh V. (2020). Robotic-Assisted First Rib Resection: Our Experience and Review of the Literature. Semin. Thorac. Cardiovasc. Surg..

[65] Gharagozloo F., Atiquzzaman N., Meyer M., Tempesta B., Werden S. (2021). Robotic first rib resection for thoracic outlet syndrome. J. Thorac. Dis..

[66] Zehnder A., Lutz J., Dorn P., Minervini F., Kestenholz P., Gelpke H., Schmid R.A., Kocher G.J. (2021). Robotic-Assisted Thoracoscopic Resection of the First Rib for Vascular Thoracic Outlet Syndrome: The New Gold Standard of Treatment?. J. Clin. Med..

[67] Gkikas A., Lampridis S., Patrini D., Kestenholz P.B., Azenha L.F., Kocher G.J., Scarci M., Minervini F. (2022). Thoracic Outlet Syndrome: Single Center Experience on Robotic Assisted First Rib Resection and Literature Review. Front. Surg..

[68] Coyan G., Daon E. (2016). Resection of supernumerary intrathoracic rib using robotic-assisted video-assisted thoracoscopic surgery. Surg. Radiol. Anat..

[69] Liu B., Gao S., Wu Q., Li H., Zhang G., Fu J. (2022). A case report of robotic-assisted resection of large fibrous benign tumor of second rib. J. Cardiothorac. Surg..

[70] Rojo M., Abdelsattar Z. (2023). Robotic resection of a second rib osteochondroma. Multimed. Man. Cardiothorac. Surg..

[71] Zehnder A., Dorn P., Lutz J., Minervini F., Kestenholz P., Gelpke H., Schmid R.A., Kocher G.J. (2022). Completely Thoracoscopic 3-Port Robotic First Rib Resection for Thoracic Outlet Syndrome. Ann. Thorac. Surg..

[72] Ureña A., Déniz C., Muñoz A., Macía I., Rivas F., Ramos R. (2023). Uniportal robotic-assisted thoracoscopic surgery: Resection of the first rib. Ann. Cardiothorac. Surg..

[73] Reyes M., Alaparthi S., Roedl J.B., Moreta M.C., Evans N.R., Grenda T., Okusanya O.T. (2023). Robotic First Rib Resection in Thoracic Outlet Syndrome: A Systematic Review of Current Literature. J. Clin. Med..

[74] Burt B.M., Palivela N., Cekmecelioglu D., Paily P., Najafi B., Lee H.-S., Montero M. (2020). Safety of robotic first rib resection for thoracic outlet syndrome. J. Thorac. Cardiovasc. Surg..

[75] Lazzaro R., Patton B. (2020). Commentary: Robotic first rib resection-Building the next pillar. JTCVS Tech..

[76] Burt B.M., Palivela N., Karimian A., Goodman M.B. (2020). Transthoracic robotic first rib resection: Twelve steps. JTCVS Tech..

[77] Palivela N.B., Burt B.M. (2023). Transthoracic Robotic First and Cervical Rib Resection for Thoracic Outlet Syndrome. Ann. Surg..

[78] Egyud M.R., Holmes S., Burt B.M. (2023). Technical Aspects of Robotic First Rib Resection. Thorac. Surg. Clin..

[79] Jiao W., Zhao Y., Luo Y., Wang H., Yang X., Ren X., Zhang L., Luo Y. (2015). Totally robotic-assisted non-circumferential tracheal resection and anastomosis for leiomyoma in an elderly female. J. Thorac. Dis..

[80] Qiu T., Zhao Y., Song J., Jiao W. (2019). Robotic circumferential tracheal resection and reconstruction via a completely portal approach. Thorac. Cancer.

[81] Li S., Ai Q., Liang H., Liu H., Yang C., Deng H., Zhong Y., Zhang J., He J. (2022). Nonintubated Robotic-assisted Thoracic Surgery for Tracheal/Airway Resection and Reconstruction: Technique Description and Preliminary Results. Ann. Surg..

[82] Zalepugas D., Schnorr P., Schmidt J., Bedetti B. (2021). Non-intubated robotic-assisted thoracic surgery for tracheal/airway resection and reconstruction safe: Editorial commentary. Ann. Transl. Med..

[83] Spaggiari L., Galetta D., Iacono G.L., Cara A., Bertolaccini L., Casiraghi M., Mohamed S. (2023). Robotic-assisted tracheal resection for adenoid cystic carcinoma with extracorporeal membrane oxygenation support. JTCVS Tech..

[84] Dal Negro R.W., Tognella S., Guerriero M., Micheletto C. (2013). Prevalence of tracheobronchomalacia and excessive dynamic airway collapse in bronchial asthma of different severity. Multidiscip. Respir. Med..

[85] Boiselle P.M., O’Donnell C.R., Bankier A.A., Ernst A., Millet M.E., Potemkin A., Loring S.H. (2009). Tracheal collapsibility in healthy volunteers during forced expiration: Assessment with multidetector CT. Radiology.

[86] Bakhos C.T., Abbas A.E. (2022). The evolution of tracheobronchoplasty. J. Vis. Surg..

[87] Tse D.G., Han S.M., Charuworn B., Kaufer E.S. (2011). Video-assisted thoracoscopic surgical tracheobronchoplasty for tracheobronchomalacia. J. Thorac. Cardiovasc. Surg..

[88] Lazar J.F., Posner D.H., Palka W., Spier L.N., Lazzaro R.S. (2015). Robotically Assisted Bilateral Bronchoplasty for Tracheobronchomalacia. Innovations.

[89] Lazzaro R., Kontopidis I., Medina B.D. (2023). Just breathe: 12-step robotic tracheobronchoplasty. JTCVS Tech..

[90] Milman S., Ng T. (2019). Robotic tracheobronchoplasty is feasible, but which patients truly benefit?. J. Thorac. Cardiovasc. Surg..

[91] Lazzaro R.S., Patton B.D., Wasserman G.A., Karp J., Cohen S., Inra M.L., Scheinerman S.J. (2022). Robotic-assisted tracheobronchoplasty: Quality of life and pulmonary function assessment on intermediate follow-up. J. Thorac. Cardiovasc. Surg..

[92] Seastedt K.P., Wilson J.L., Gangadharan S.P. (2023). Robotic Surgery for Tracheobronchomalacia. Thorac. Surg. Clin..

[93] Inra M.L., Wasserman G.A., Karp J., Cohen S., Scheinerman S.J., Lazzaro R.S. (2023). Improvement in postoperative lung function in patients with moderate to severe airway obstruction after robotic-assisted thoracoscopic tracheobronchoplasty. J. Thorac. Cardiovasc. Surg..

[94] Kadiyala M., Maxfield M.W., Uy K.F., Blankenship D., Adler A.C. (2022). Successful Use of an EZ-blocker for Lung Isolation and Visualization of Sutures During Minimally Invasive Robotic Tracheobronchoplasty in a Patient With Difficult Airway. J. Cardiothorac. Vasc. Anesth..

[95] Groth S.S.M.D., Andrade R.S.M.D. (2009). Diaphragmatic Eventration. Thorac. Surg. Clin..

[96] Taberham R.J., Raza A., Alzetani A., Woo E.B., Chamberlain M.H., Koulaxouzidis G., Amer K.M. (2017). VATS Plication of the Diaphragm: A Descriptive Observational 10-Year Southampton Experience. Innovations.

[97] Nardini M., Jayakumar S., Migliore M., Nosotti M., Paul I., Dunning J. (2021). Minimally Invasive Plication of the Diaphragm: A Single-Center Prospective Study. Innov. Technol. Tech. Cardiothorac. Vasc. Surg..

[98] Hunt A.R.B., Stuart C.M., Gergen A.K., Bang T.J., Reihman A.E., Helmkamp L.J., Lin Y., Mitchell J.D., Meguid R.A., Scott C.D. (2023). Long-Term Patient-Reported Symptom Improvement and Quality of Life after Transthoracic Diaphragm Plication in Adults. J. Am. Coll. Surg..

[99] Kwak T., Lazzaro R., Pournik H., Ciaburri D., Tortolani A., Gulkarov I. (2011). Robotic thoracoscopic plication for symptomatic diaphragm paralysis. J. Robot. Surg..

[100] Ahn J., Suh J., Jeong J. (2013). Robot-assisted thoracoscopic surgery with simple laparoscopy for diaphragm eventration. Thorac. Cardiovasc. Surg..

[101] Counts S.J., Saffarzadeh A.G., Blasberg J.D., Kim A.W. (2018). Robotic Transthoracic Primary Repair of a Diaphragmatic Hernia and Reduction of an Intrathoracic Liver. Innovations.

[102] Daniels A., Danau T., Chierchia G.B., de Asmundis C., Lamote J., Smets D. (2019). Robot-assisted thoracoscopic diaphragm plication for symptomatic diaphragm paralysis after cryoballoon ablation. Hear. Case Rep..

[103] Schumacher L., Zhao D. (2021). Outcomes and technique of robotic diaphragm plication. J. Thorac. Dis..

[104] Hurley P., Djouani A., Lampridis S., Bille A. (2022). Diaphragmatic paralysis post COVID-19 treated with robot-assisted plication reinforced with acellular dermal matrix: A report of two cases. Monaldi Arch. Chest Dis..

[105] Gergen A.K., Stuart C.M., Wojcik B.M., Meguid R.A., Scott C.D. (2023). Robotic-Assisted Transthoracic Diaphragm Plication. Oper. Tech. Thorac. Cardiovasc. Surg..

[106] Xu P.P., Chang X.P., Tang S.T., Li S., Cao G.Q., Zhang X., Chi S.Q., Fang M.J., Yang D.H., Li X.Y. (2020). Robot-assisted thoracoscopic plication for diaphragmatic eventration. J. Pediatr. Surg..

[107] Biswas Roy S., Haworth C., Ipsen T., Kang P., Hill D., Do A., Kuo E. (2018). Transabdominal robot-assisted diaphragmatic plication: A 3.5-year experience. Eur. J. Cardio-Thorac. Surg..

[108] Bin Asaf B., Kodaganur Gopinath S., Kumar A., Puri H.V., Pulle M.V., Bishnoi S. (2021). Robotic diaphragmatic plication for eventration: A retrospective analysis of efficacy, safety, and feasibility. Asian J. Endosc. Surg..

[109] Zwischenberger B.A., Kister N., Zwischenberger J.B., Martin J.T. (2016). Laparoscopic Robot-Assisted Diaphragm Plication. Ann. Thorac. Surg..

[110] Du V.X., Groth S.S. (2022). Robot-Assisted Laparoscopic Diaphragm Plication. Oper. Tech. Thorac. Cardiovasc. Surg..

[111] Gritsiuta A.I., Gordon M., Bakhos C.T., Abbas A.E., Petrov R.V. (2022). Minimally Invasive Diaphragm Plication for Acquired Unilateral Diaphragm Paralysis: A Systematic Review. Innov. Technol. Tech. Cardiothorac. Vasc. Surg..

[112] Melvin W.S., Needleman B.J., Krause K.R., Schneider C., Wolf R.K., Michler R.E., Ellison E.C. (2002). Computer-enhanced robotic telesurgery. Initial experience in foregut surgery. Surg. Endosc..

[113] Kernstine K.H., DeArmond D.T., Karimi M., Van Natta T.L., Campos J.H., Yoder M.R., Everett J.E. (2004). The robotic, 2-stage, 3-field esophagolymphadenectomy. J. Thorac. Cardiovasc. Surg..

[114] Hoelzen J.P., Frankauer B.E., Szardenings C., Roy D., Pollmann L., Fortmann L., Merten J., Rijcken E., Juratli M.A., Pascher A. (2023). Reducing the Risks of Esophagectomies: A Retrospective Comparison of Hybrid versus Full-Robotic-Assisted Minimally Invasive Esophagectomy (RAMIE) Approaches. J. Clin. Med..

[115] Watanabe M., Kuriyama K., Terayama M., Okamura A., Kanamori J., Imamura Y. (2023). Robotic-Assisted Esophagectomy: Current Situation and Future Perspectives. Ann. Thorac. Cardiovasc. Surg..

[116] Weindelmayer J., De Pasqual C.A., Turolo C., Gervasi M.C., Sacco M., Bencivenga M., Giacopuzzi S. (2023). Robotic versus open Ivor-Lewis esophagectomy: A more accurate lymph node dissection without burdening the leak rate. J. Surg. Oncol..

[117] Ekeke C.N., Kuiper G.M., Luketich J.D., Ruppert K.M., Copelli S.J., Baker N., Levy R.M., Awais O., Christie N.A., Dhupar R. (2023). Comparison of robotic-assisted minimally invasive esophagectomy versus minimally invasive esophagectomy: A propensity-matched study from a single high-volume institution. J. Thorac. Cardiovasc. Surg..

[118] van der Sluis P.C., van der Horst S., May A.M., Schippers C., Brosens L.A.A., Joore H.C.A., Kroese C.C., Haj Mohammad N., Mook S., Vleggaar F.P. (2019). Robot-assisted Minimally Invasive Thoracolaparoscopic Esophagectomy Versus Open Transthoracic Esophagectomy for Resectable Esophageal Cancer: A Randomized Controlled Trial. Ann. Surg.

[119] Khaitan P.G., Vekstein A.M., Thibault D., Kosinski A., Hartwig M.G., Block M., Gaissert H., Wolf A.S. (2023). Robotic Esophagectomy Trends and Early Surgical Outcomes: The US Experience. Ann. Thorac. Surg..

[120] Till B.M., Grenda T.R., Okusanya O.T., Evans Iii N.R. (2023). Robotic Minimally Invasive Esophagectomy. Thorac. Surg. Clin..

[121] Knitter S., Maurer M.M., Winter A., Dobrindt E.M., Seika P., Ritschl P.V., Raakow J., Pratschke J., Denecke C. (2023). Robotic-Assisted Ivor Lewis Esophagectomy Is Safe and Cost Equivalent Compared to Minimally Invasive Esophagectomy in a Tertiary Referral Center. Cancers.

[122] Dunnican W.J., Singh T.P., Guptill G.G., Doorly M.G., Ata A. (2008). Early robotic experience with paraesophageal hernia repair and Nissen fundoplication: Short-term outcomes. J. Robot. Surg..

[123] DeUgarte D.A., Hirschl R.B., Geiger J.D. (2009). Robotic Repair of Congenital Paraesophageal Hiatal Hernia. J. Laparoendosc. Adv. Surg. Tech..

[124] Fu S.S., Carton M.M., Ghaderi I., Galvani C.A. (2018). Robotic-Assisted Simultaneous Repair of Paraesophageal Hernia and Morgagni Hernia: Technical Report. J. Laparoendosc. Adv. Surg. Tech..

[125] Gehrig T., Mehrabi A., Fischer L., Kenngott H., Hinz U., Gutt C.N., Muller-Stich B.P. (2013). Robotic-assisted paraesophageal hernia repair—A case-control study. Langenbeck’s Arch. Surg..

[126] Galvani C.A., Loebl H., Osuchukwu O., Samamé J., Apel M.E., Ghaderi I. (2016). Robotic-Assisted Paraesophageal Hernia Repair: Initial Experience at a Single Institution. J. Laparoendosc. Adv. Surg. Tech..

[127] Vasudevan V., Reusche R., Nelson E., Kaza S. (2018). Robotic paraesophageal hernia repair: A single-center experience and systematic review. J. Robot. Surg..

[128] Gerull W.D., Cho D., Kuo I., Arefanian S., Kushner B.S., Awad M.M. (2020). Robotic Approach to Paraesophageal Hernia Repair Results in Low Long-Term Recurrence Rate and Beneficial Patient-Centered Outcomes. J. Am. Coll. Surg..

[129] Sarkaria I.S., Latif M.J., Bianco V.J., Bains M.S., Rusch V.W., Jones D.R., Rizk N.P. (2017). Early operative outcomes and learning curve of robotic assisted giant paraesophageal hernia repair. Int. J. Med. Robot. Comput. Assist. Surg..

[130] Bhatt H., Wei B. (2023). Comparison of laparoscopic vs. robotic paraesophageal hernia repair: A systematic review. J. Thorac. Dis..

[131] Lekarczyk A., Sinha H., Dvir D., Goyert J., Airhart A., Reddy R.M. (2023). Similar hospital profits with robotic-assisted paraesophageal hiatal hernia repair, despite higher or supply costs. Surg. Endosc..

[132] Panse N.S., Prasath V., Quinn P.L., Chokshi R.J. (2023). Economic evaluation of robotic and laparoscopic paraesophageal hernia repair. Surg. Endosc..

[133] Sowards K.J., Holton N.F., Elliott E.G., Hall J., Bajwa K.S., Snyder B.E., Wilson T.D., Mehta S.S., Walker P.A., Chandwani K.D. (2020). Safety of robotic assisted laparoscopic recurrent paraesophageal hernia repair: Insights from a large single institution experience. Surg. Endosc..

[134] Tartaglia N., Pavone G., Di Lascia A., Vovola F., Maddalena F., Fersini A., Pacilli M., Ambrosi A. (2020). Robotic voluminous paraesophageal hernia repair: A case report and review of the literature. J. Med. Case Rep..

[135] Gerull W.D., Cho D., Arefanian S., Kushner B.S., Awad M.M. (2021). Favorable peri-operative outcomes observed in paraesophageal hernia repair with robotic approach. Surg. Endosc..

[136] Tonelli C.M., Baker M.S., Luchette F.A., Cohn T. (2023). Laparoscopic and robotic paraesophageal hernia repair in United States veterans: Clinical outcomes and risk factors associated with reoperation recurrence. Am. J. Surg..

[137] Reza J.A., Bakhos C., Su S., Petrov R., Abbas A.E. (2023). Robotic Belsey Mark IV Repair of the Paraesophageal Hernia. Innov. Technol. Tech. Cardiothorac. Vasc. Surg..

[138] Manfredini B., Zirafa C.C., Romano G., Bagalà E., Cariello C., Davini F., Melfi F. (2023). Intraoperative Catastrophes during Robotic Lung Resection: A Single-Center Experience and Review of the Literature. Life.

[139] Sakakura N., Nakada T., Shirai S., Takahara H., Suzuki A., Takahashi Y., Kuroda H. (2022). Emergency rollout and conversion procedures during the three-arm robotic open-thoracotomy-view approach. Interact. Cardiovasc. Thorac. Surg..

[140] Nelson D.B., Mehran R.J., Mitchell K.G., Rajaram R., Correa A.M., Bassett R.L., Antonoff M.B., Hofstetter W.L., Roth J.A., Sepesi B. (2019). Robotic-Assisted Lobectomy for Non-Small Cell Lung Cancer: A Comprehensive Institutional Experience. Ann. Thorac. Surg..

[141] Rogers M.P., Janjua H., Eguia E., Lozonschi L., Toloza E.M., Kuo P.C. (2022). Adopting robotic thoracic surgery impacts hospital overall lung resection case volume. Am. J. Surg..

[142] Harrison O.J., Maraschi A., Routledge T., Lampridis S., LeReun C., Bille A. (2023). A cost analysis of robotic vs. video-assisted thoracic surgery: The impact of the learning curve and the COVID-19 pandemic. Front. Surg..

[143] Shanahan B., Kreaden U.S., Sorensen J., Stamenkovic S., Redmond K.C. (2022). Is robotic lobectomy cheaper? A micro-cost analysis. J. Robot. Surg..

[144] Bellini V., Valente M., Del Rio P., Bignami E. (2021). Artificial intelligence in thoracic surgery: A narrative review. J. Thorac. Dis..

